# The complete mitochondrial genome of *Trichochrysea japana* (Motschulsky, 1855) (Coleoptera: Chrysomeloidea) and its phylogenetic analyses

**DOI:** 10.1080/23802359.2025.2487217

**Published:** 2025-04-02

**Authors:** Lulu Huang, Hang Zhang, Ying Chen, Xinquan Li, Jiankai Wu, Songqing Wu

**Affiliations:** a College of Forestry, Fujian Agriculture and Forestry University, Fuzhou, China; b Key Laboratory of Integrated Pest Management in Ecological Forests, Fujian Province University, Fujian Agriculture and Forestry University, Fuzhou, China; c Fujian Provincial Forestry Science and Technology Extension Station, Fuzhou, China

**Keywords:** *Trichochrysea japana*, mitogenome, phylogenetic analysis

## Abstract

*Trichochrysea japana* (Motschulsky, 1855) (Coleoptera: Chrysomelidae) is a horticultural pest with a widespread distribution. However, the complete mitochondrial genome (mitogenome) of this species has not been available . In this study, we sequenced and analyzed the first complete mitogenome of *T. japana*. The mitogenome is 15,681 bp in length (GenBank accession number: OR387477) and includes 13 typicalPCGs, 22 tRNAs, two rRNAs, and two control regions. Furthermore, phylogenetic analysis indicated that *T. japana* is closely related to *Basilepta melanopus*, *B. fulvipes*, and *Colasposoma dauricum*. The *T. japana* mitogenome data provides valuable insights into the genetic evolution of this species .

## 1. Introduction

**Figure 1.**
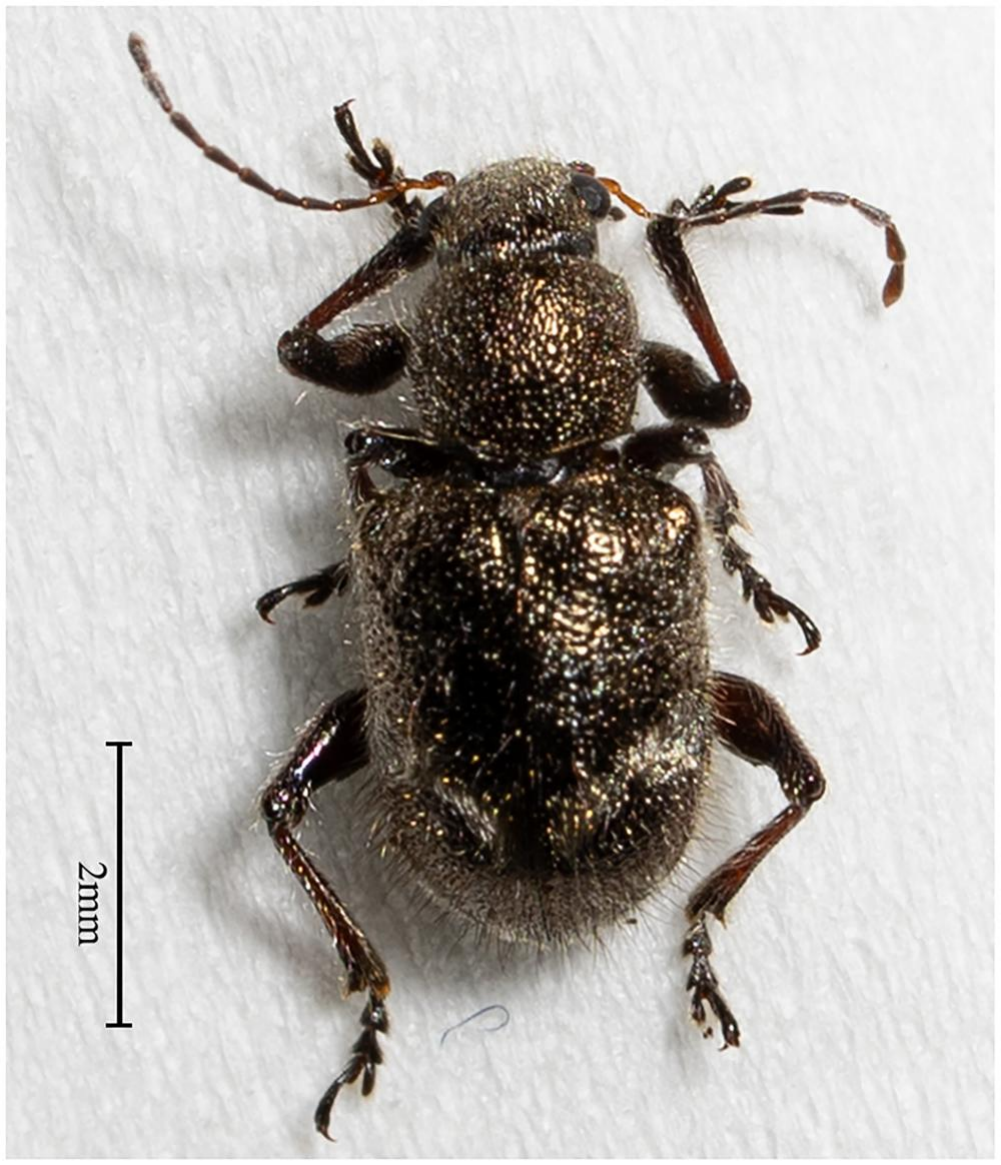
Morphological photograph of *Trichochrysea japana* (photographed by Lulu Huang at Fujian Agriculture and Forestry University, Fuzhou City, China).

*Trichochrysea japana* (Motschulsky, 1855), a member of the Chrysomelidae family within the Coleoptera order, has a wide geographical distribution, spanning China, Japan, and Korea (Komiya 1985). Notably, it is commonly found across several provinces in China, including Fujian, Hunan, Anhui, Guangxi, and Taiwan, where it poses a significant threat to tea trees (Zhang 1999). Hunan, renowned for its copious oil tea tree resources, is particularly vulnerable to the harmful effects of *T. japana* on tea tree growth (Li et al. 2013). The superfamily Chrysomeloidea, which encompasses seven distinct families, further highlights the ecological importance and potential challenges posed by this diverse insect group (Mckenna et al. 2015). Once the adults emerge from the soil, they feed on leaves and buds, weakening the host plant and making it more susceptible to various diseases (He et al. 2010). The body of *T. japana* is oval-shaped, brown, and about eight millimeters long. It has green spots on its wings, black hairs on its body, elongated antennae, and distinct internodes (Figure 1).

Recent advances in molecular biology have greatly improved insect classification systems and comprehensive mitogenome analysis has provided valuable insights into the genetics and developmental patterns of species (Zhang 2006). This study analyzed the mitogenome characteristics and phylogenetic placement of *T. japana*, offering essential insights into its genetic and evolutionary relationships.

## 2. Materials

*T. japana* was discovered in Lianjiang County, Fuzhou City, Fujian Province, China (119°23′38′′E, 26°3′7′′N). The specimen voucher, designated as YJ-202301, is securely stored in the laboratory of the College of Forestry at Fujian Agriculture and Forestry University (Songqing Wu, dabinyang@126.com).

## 3. Methods

### 3.1. Genomic DNA extraction and sequencing

The leg of an adult *T. japana* was used as the sample, and total genomic DNA was extracted using the TruSeq DNA sample preparation kit (Vazyme, Fuzhou, China) and purified with the QIAquick Gel Extraction kit (Qiagen, Hilden, Germany). The concentration and purity of the extracted DNA were accurately assessed using a Nanodrop 2000 spectrophotometer (Thermo Fisher Scientific, Massachusetts, USA). The samples were sequenced on the Illumina HiSeq 2500 platform, employing a paired-end sequencing strategy with 2 × 150 bp reads.

### 3.2. Mitogenome assembly and annotation

Low-quality reads and artifacts were removed from the original dataset, which contained a total of 49,598,828 reads, resulting in 887,692 clean reads. These clean reads were assembled using MitoZ (Meng et al. 2019) and metaSPAdes software (Nurk et al. 2017). Genome annotation was performed using mitoMaker software (Prosdocimi and Schomaker-Bastos 2018). To ensure accuracy, the complete mitogenome sequence of *T. japana* was corrected regarding *Platycorynus* sp. N26 (GenBank accession number: MK049872.1) (Nie et al. 2020b).

### 3.3. Phylogenetic analysis

To analyze the taxonomic status of *T. japana* more comprehensively, we constructed a phylogenetic tree using the mitogenome of *T. japana* and other 22 Chrysomeloidea species as the ingroup, with *Dinapate wrightii* (Coleoptera: Bostrichoidea; GenBank accession number: ON707241.1) was used as outgroup (Table 1). The complete mitogenome sequences of all species were aligned using MEGA7 software (Katoh and Standley 2013). The phylogenetic tree was then generated using the maximum-likelihood (ML) method within the IQ-TREE software, with 1000 bootstrap replicates (Nguyen et al. 2015). Finally, the phylogenetic tree was visualized using iTol v6 (https://itol.embl.de/).

**Table 1.** Information about the mitochondrial genome of the species used in this study.

Family	Subfamily	Species	GenBank no.	References
Chrysomelidae	Eumolpinae	*Trichochrysea japana*	OR387477	This study
		*Colasposoma dauricum*	NC_057218.1	Unpublished
		*Colasposoma dauricum*	KY039104.1	(Song et al. 2018)
		*Basilepta fulvipes*	NC_054189.1	(Liu et al. 2020)
		*Basilepta melanopus*	NC_066974.1	Unpublished
		*Basilepta melanopus*	OP115728.2	Unpublished
		*Aoria nigripes*	NC_065028.1	Unpublished
		*Bromius obscurus*	KX087249.1	Unpublished
		*Colasposoma dauricum*	OR502857.1	Unpublished
		*Colasposoma dauricum*	MW528208.1	Unpublished
		*Platycorynus* sp. N26	MK049872.1	(Nie et al. 2020b)
	Galerucinae	*Monolepta hieroglyphica*	OL504952.1	Unpublished
		*Aoria nigripes*	KX943504.1	(Gómez‐Rodríguez et al. 2015)
Cerambycidae	Lamiinae	*Monochamus alternatus*	NC_024652.1	(Li et al. 2016)
		*Monochamus alternatus*	MW858152.1	(Zhang et al. 2021)
		*Monochamus alternatus*	JX987292.1	(Wang et al. 2013)
		*Monochamus sartor urussovii*	OP169420.1	(Shi et al. 2023)
		*Oberea diversipes*	NC_053945.1	Unpublished
		*Agelasta perplexa*	NC_053905.1	Unpublished
	Lepturinae	*Stictoleptura palmi*	OQ716389.1	Unpublished
		*Rhagium fortecostatum*	MN473103.1	(Nie et al. 2020a)
	Aseminae	*Cephalallus oberthueri*	NC_062854.1	Unpublished
		*Arhopalus unicolor*	NC_053904.1	Unpublished
Bostrichidae	Bostrichinae	*Dinapate wrightii*	ON707241.1	Unpublished

## 4. Results

**Figure 2.**
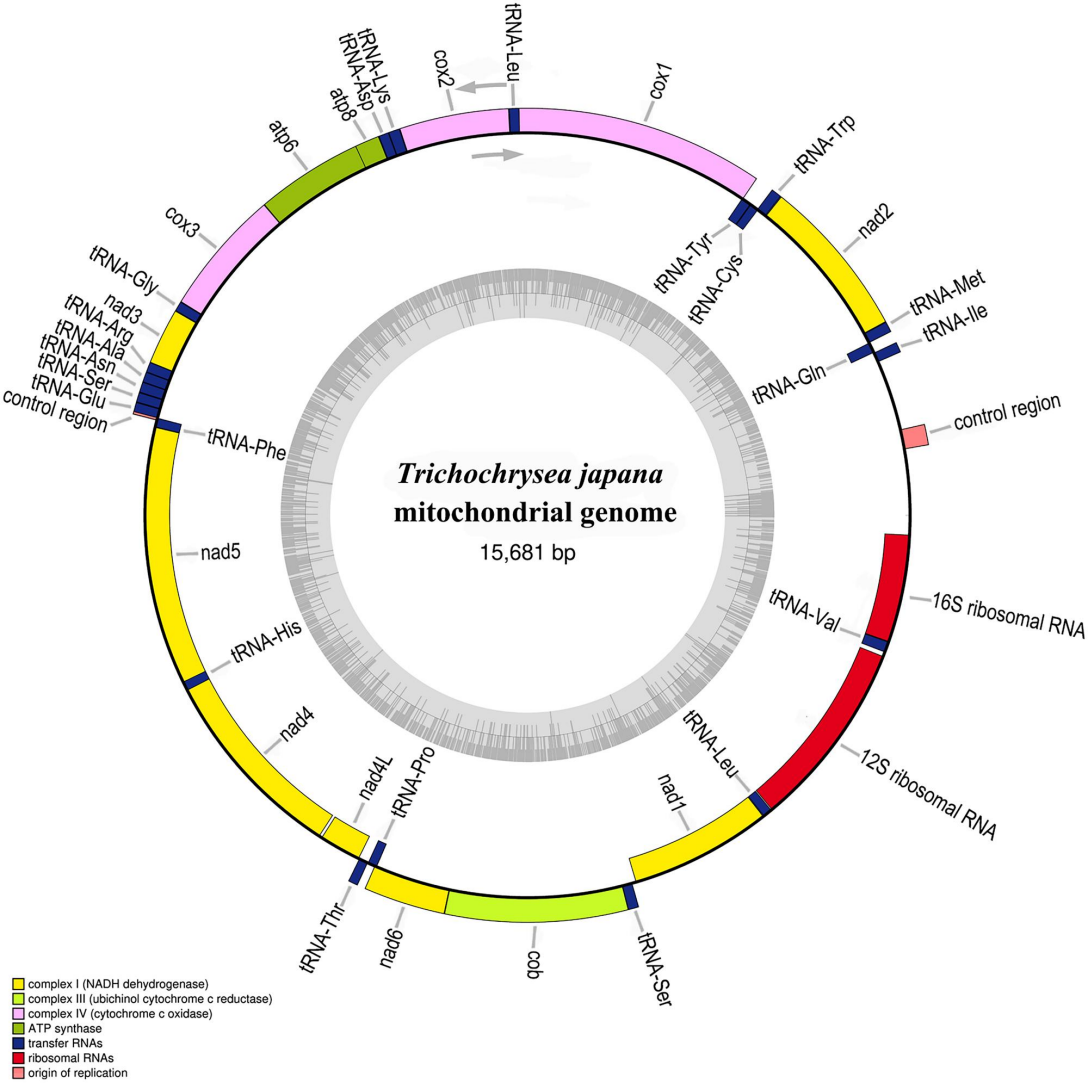
Mitochondrial genome map of the *Trichochrysea japana* (GenBank: OR387477) the gene’s GC content is shown by gray inner circles, different gene functions are represented by colored outside circles, and transcriptional direction is represented by arrows.

The mitogenome of *T. japana* was constructed using high-coverage sequencing data, with a coverage rate exceeding 5000 × (Figure S1). The total length of the *T. japana* mitogenome is 15,681 bp (GenBank accession number: OR387477), with a GC content of 35.2%, consisting of 41.6% adenine (A), 8.6% guanine (G), 14% cytosine (C), and 35.8% thymine (T). The complete mitogenome contains 13 protein-coding genes (PCGs), 22 transfer RNAs (tRNAs), two ribosomal RNAs (rRNAs), and two control regions (Figure 2). Fourteen genes are transcribed from the minority strand (N-strand), including eight tRNAs, four PCGs, and two rRNAs, while the remaining fourteen tRNAs and nine PCGs are transcribed from the majority strand (J-strand). The lengths of the 12S and 16S rRNAs are 1251 bp and 714 bp, respectively. The 22 tRNAs molecules in this genome assembly ranged from 61 bp (tRNA-Cys) to 73 bp (tRNA-Val). The total length of the 13 PCGs is 11,111 bp, encoding 3710 amino acids. Most PCGs begin with standard start codons ATD (ATT, ATG, ATA), with the exceptions of *nad4* and *nad5*, which use TAT; *nad4L*, which starts with CAT; and *nad1, which* begins with CAA. The termination codons for the PCGs include TAA, GCT, TAG, ATT, and an incomplete codon (T).

**Figure 3.**
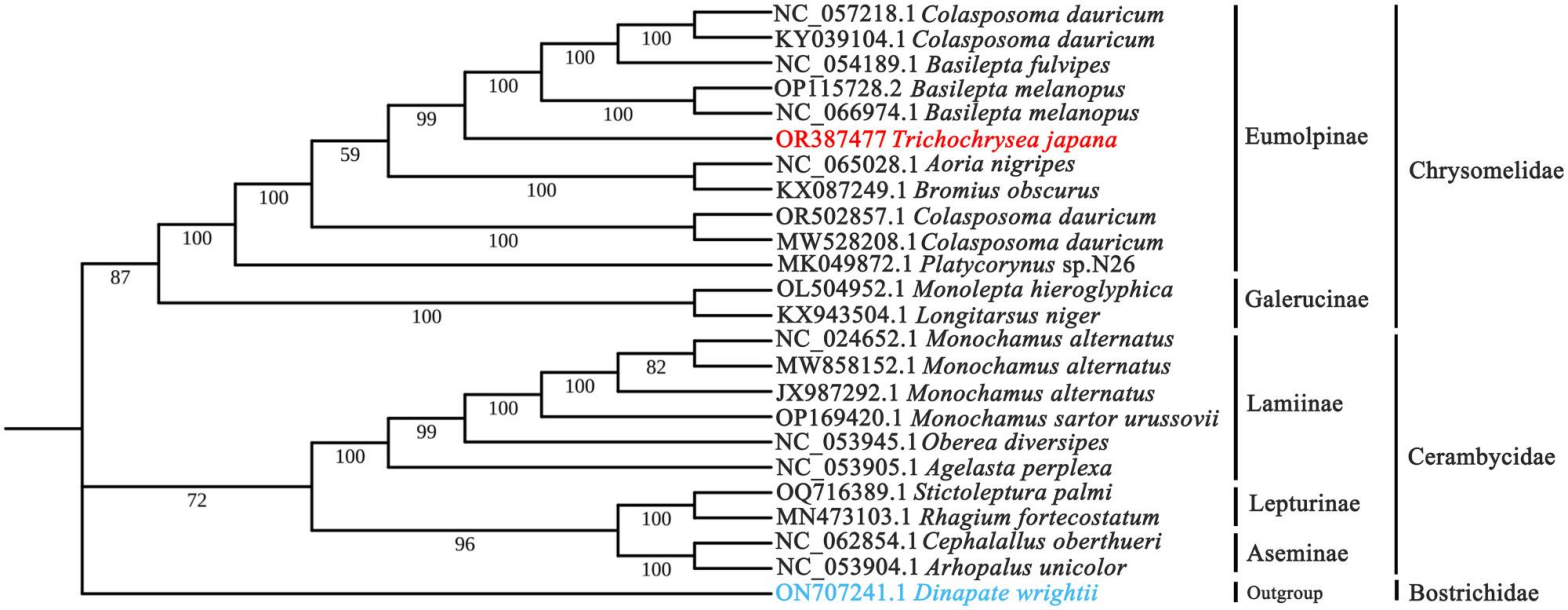
Maximum-likelihood analysis of *Trichochrysea japana* and 23 related species insects based on genome sequence with 1000 bootstraps. Bootstrap support values are labeled near the branch. The species analyzed in this study are represented by red fonts, and the outgroup is represented by blue fonts. The sequences from the following species were used: *Trichochrysea japana* OR387477 (This study), *Colasposoma dauricum* NC_057218.1, *Colasposoma dauricum* KY039104.1 (Song et al. 2018), *Basilepta fulvipes* NC_054189.1 (Liu et al. 2020), *Basilepta melanopus* NC_066974.1, *Basilepta melanopus* OP115728.2, *Aoria nigripes* NC_065028.1, *Bromius obscurus* KX087249.1, *Colasposoma dauricum* OR502857.1, *Colasposoma dauricum* MW528208.1, *Platycorynus* sp. N26 MK049872.1 (Nie et al. 2020b) *Monolepta hieroglyphica* OL504952.1, *Aoria nigripes* KX943504.1 (Gómez‐Rodríguez et al. 2015), *Monochamus alternatus* NC_024652.1 (Li et al. 2016), *Monochamus alternatus* MW858152.1 (Zhang et al. 2021), *Monochamus alternatus* JX987292.1 (Wang et al. 2013), *Monochamus sartor urussovii* OP169420.1 (Shi et al. 2023), *Oberea diversipes* NC_053945.1, *Agelasta perplexa* NC_053905.1, *Stictoleptura palmi* OQ716389.1, *Rhagium fortecostatum* MN473103.1 (Nie et al. 2020a), *Cephalallus oberthueri* NC_062854.1, *Arhopalus unicolor* NC_053904.1, *Dinapate wrightii* ON707241.1.

Phylogenetic analysis showed that the analyzed species were clustered into three main branches. The first branch, located at the root of the tree, consists of *D. wrightii*, which belongs to the Bostrichoidea superfamily within Coleoptera. The second branch includes 10 species from the Lamiinae, Lepturinae, and Aseminae subfamilies of the Cerambycidae family. The third branch contains 13 species, including *T. japana*, which belongs to the Chrysomelidae of Coleoptera. Within this branch, *T. japana* is closely related to *B. melanopus*, *B. fulvipes,* and *C. dauricum* and is classified within the subfamily Eumolpinae (Figure 3).

## 5. Discussion and conclusion

In this study, the mitogenome of *T. japana* was successfully sequenced and annotated, revealing a comprehensive mitogenome containing 37 genes, similar to the organization observed in the hypothetical ancestral insects (Clary and Wolstenholme 1985). The presence of multiple control regions in the mitogenome of *T. japana* and other select insects is likely due to evolutionary gene mutations (Wei and Chen 2011). *T. japana* displays a characteristic bias toward high A + T content (A + *T* = 77.4%, G + *C* = 22.6%), with the combined proportions of A and C being higher than those of T and G (A + *C* = 55.6%, T + *G* = 44.4%), consistent with patterns observed in other insects (Raupach et al. 2022). Furthermore, the phylogenetic analysis provides essential insights into the evolutionary dynamics of the mitogenome in this species and offers an indispensable foundation for future genetic research. Additionally, acquiring *T. japana* mitogenome data will significantly aid the development of new biological control strategies for managing this pest.

## Supplementary Material

Supplementary Material.docx

## Data Availability

The genome sequence data supporting this study’s findings are available in GenBank of NCBI at (https://www.ncbi.nlm.nih.gov) under assessment no. OR387477. The associated BioProject, Bio-Sample, and SRA numbers were PRJNA1050016, SAMN38724401, and SRR27142794. The RefSeq BioProjects corresponding to this sample is PRJNA927338. This page contains information about the role of RefSeq BioProjects: https://www.ncbi.nlm.nih.gov/refseq/about/.
