# Supplementary material for: The complete mitochondrial genome of Trichochrysea japana (Motschulsky, 1855) (Coleoptera: Chrysomeloidea) and its phylogenetic analyses
Source: Mitochondrial DNA B Resour. 2025 Apr 2;10(5):352–6. doi: 10.1080/23802359.2025.2487217 (PMC11966972; doi:10.1080/23802359.2025.2487217)
Supplement: Supplementary Material.docx [file TMDN_A_2487217_SM1010.docx]

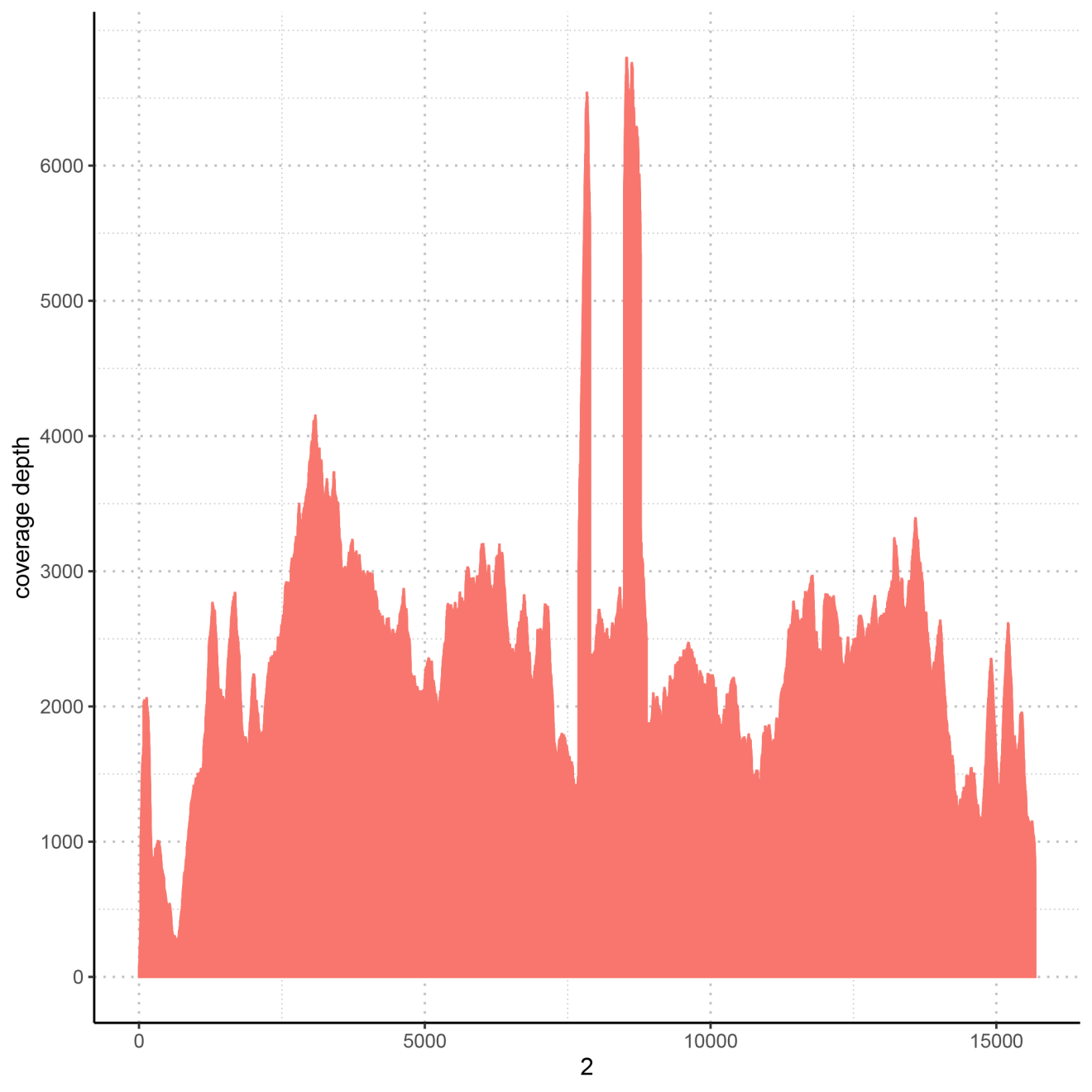


**Figure S1.** Depth of coverage for *Trichochrysea japana* mitochondrial genome. X and Y axis present nucleotide position of *T. japana* mitochondrial genome and coverage depth, respectively.
